# Human Rights Treaties Are an Important Part of the "International Health Instrumentariam": Comment on "The Legal Strength of International Health Instruments - What It Brings to Global Health Governance?"

**DOI:** 10.15171/ijhpm.2017.109

**Published:** 2017-10-02

**Authors:** Lisa Forman

**Affiliations:** Dalla Lana School of Public Health, University of Toronto, Toronto, ON, Canada.

**Keywords:** Human Rights, International Law, Right to Health, Global Health

## Abstract

In their commentary, Haik Nikogosian and Ilona Kickbusch argue for the necessity of new binding international legal instruments for health to address complex health determinants and offer a cogent analysis of the implications of such treaties for future global health governance. Yet in doing so they pay no attention to the existing instrumentarium of international legally binding treaties relevant to health, in the form of human rights treaties. International human rights law has entrenched individual entitlements and state obligations in relation to individual and public health through iterative human rights treaties since 1946. These treaties offer normative specificity, institutional monitoring and the possibility of enforcement and accountability. If we are to build a new ‘international health instrumentariam’ we should not ignore existing and important tools that can assist in this endeavor.

Haik Nikogosian and Ilona Kickbusch’s recent *IJHPM* article argues that “[i]nternational legal instruments for health, with their binding character and strength, have a special place” in the development of public health instruments capable of addressing increasingly complex health determinants.^1^ They argue that instruments like the World Health Organization’s (WHO’s) Framework Convention on Tobacco Control and the new Protocol to Eliminate Illicit Trade in Tobacco Products **“**opened a new phase in WHO-era global health that accepted international legally binding treaties as one major way forward.”^1^ Their article offers a cogent and important analysis of the implications of these new health treaties current and for future global health governance. One might assume reading this commentary that international law has lacked an instrumentarium of international legally binding treaties relevant to health until these recent instruments.

Yet international human rights law has entrenched individual entitlements and states obligations in relation to individual and public health at least since the 1946 Constitution of the WHO. The WHO Constitution recognized that “the enjoyment of the highest attainable standard of health is a fundamental right of every human being without distinction of race, religion, political belief, economic or social condition,” and that governments have a responsibility “for the health of their peoples which can be fulfilled only by the provision of adequate health and social measures.”^2^

The concept of state responsibilities in relation to health and medical care is expanded in article 25.1 of the *Universal Declaration of Human Rights* (UDHR) which holds that “everyone has the right to a standard of living adequate for the health and well-being of himself and of his family, including food, clothing, housing and medical care and necessary social services.”^3^ These state duties are articulated most authoritatively in article 12 of the 1966 *International Covenant on Economic, Social and Cultural Rights,* where states recognize the right of everyone to the enjoyment of the highest attainable standard of physical and mental health and undertake steps to realize this standard, including reducing the stillbirth rate and infant mortality; improving all aspects of environmental and industrial hygiene; preventing, treating, and controlling epidemic, endemic, occupational, and other diseases; and creating conditions that assure medical services and attention to all in the event of sickness.^4^ Additional human rights treaties entrench state duties relevant to health for vulnerable groups including racial minorities,^5^ women,^6^ children,^7^ and people with disabilities.^8^

More explicit state duties in relation to these right to health provisions have been authoritatively interpreted, including requirements that states ensure functioning public health and healthcare facilities, goods, services and programmes that are available, accessible, acceptable and of good quality.^9^ Other state duties include (to name but a few): ensuring access to family planning, pre- and post-natal care and emergency obstetric services,^9^ requiring the establishment of prevention and education programmes for behaviour-related health concerns such as sexually transmitted diseases, in particular HIV/AIDS, and those adversely affecting sexual and reproductive health, and requiring “the creation of a system of urgent medical care in cases of accidents, epidemics and similar health hazards, and the provision of disaster relief and humanitarian assistance in emergency situations.”^9^

All of these human rights treaties are subject to institutional monitoring and sometimes quasi-judicial enforcement.^10^ The treaties relating to economic, social and cultural rights, disability, women’s rights and racial discrimination allow individual complaints alleging the violation of treaty rights to be lodged against states at the relevant treaty committee. Decisions emerging from such complaints have set important normative standards and influenced national policy and law: In a 1977 decision, the United Nations (UN) Human Rights Committee found the Canadian government had violated a complainant’s rights under the International Convention on Civil and Political Rights by legally requiring that women lose their aboriginal status when marrying a non-aboriginal man (a consequence which was not applied to aboriginal men). This decision resulted in the Canadian government’s amendment of the law in question to eliminate its gender discrimination and restore aboriginal status to women previously affected.^11^ More recently in 2011, in the case of a woman who died in childbirth, the UN Committee on the Elimination of Discrimination against Women found Brazil in violation of its treaty obligation of non-discrimination for failing to assure appropriate maternal health services for all.^12^ The significance of the Pimentel decision is (at least) two-fold: Firstly, it is the first UN treaty body decision finding a preventable maternal death to be a human rights violation; Secondly, the CEDAW (Convention on the Elimination of All Forms of Discrimination against Women) decision resulted in a national judicial decision awarding civil damages, albeit without finding the state directly responsible for violations that occurred in a private health care clinic.^13^ The CEDAW Committee negotiated this award with the Brazilian government as a direct follow up to the Pimental decision.^14^

All of these human rights treaties have significantly influenced the passing of closely related domestic legislation, with over one hundred domestic constitutions entrenching health rights.^15^ These legal factors have combined to produce growing number of right to health claims at the national level that have significantly influenced national health law, policy and programs.^16,17^

So why the large gap in Nikogosian and Kickbusch’s commentary in relation to international human rights law? Is this elision of the treaties, institutions and impacts of international human rights law an implicit commentary on their perceptions of the efficacy of international human rights law treaties relevant to health? Or is it because they do not see these treaties as relevant to explicitly health-focused treaties like the Framework Convention on Tobacco Control (FCTC)? Certainly the FCTC offers an interesting case-study with respect to the links between health and human rights treaties: While its preamble explicitly acknowledges the right to health in the International Covenant on Economic, Social and Cultural Rights (ICESCR), CEDAW and the Convention on the Rights of the Child (CRC),^18^ it is notable that these references are non-binding.^19^ Moreover, a senior participant in FCTC negotiations indicates that there was little “meaningful discourse on the intersection between human rights and public health.”^20^ Despite the relative paucity of human rights within the FTCT text, domestic courts have made this linkage: for example, in a 2010 decision, the Guatemalan Constitutional Court upheld a law prohibiting public smoking on the basis of the government’s obligation to protect rights to health and life, explicitly linking both human rights treaties and FCTC in doing so.^19^ In this way, Cabrera and Gostin argue that human rights and health treaties like the FCTC can be mutually reinforcing frameworks that both shape each other’s normative content and assist in enforcement at the domestic level, including through the involvement of the UN international human rights committees.^19^

These existing and potential linkages between human rights treaties and current and prospective health treaties illustrate the imperative and opportunities for ensuring a greater integration between old and new international legal regimes related to health. A new global health treaty should adopt as an explicit mandate, the imperative to build linkages to international human rights treaties, mechanisms and actors that go far beyond the nascent mentions of the right to health in the FCTC. Possibilities include ensuring explicit linkages in a new treaty between its provisions and binding duties under ICESCR and other human rights treaties; linking the enforcement and monitoring of the new regime to existing international treaty bodies and mechanisms such as the Committee on Economic, Social and Cultural Rights’ reporting and individual complaints mechanism and the UN Special Rapporteur on the Right to Health; and assuring the participation of civil society in the drafting and the monitoring of a new treaty. In the absence of doing so, building a new ‘international health instrumentariam’ that by-passes vast aspects of the old, needlessly ignores existing and important tools in the broader policy toolkit and risks building a new edifice on incomplete foundations.

## Ethical issues

Not applicable.

## Competing interests

Author declares that she has no competing interests.

## Author’s contribution

LF is the single author of the paper.
